# Post-exercise Cold Water Immersion Does Not Improve Subsequent 4-km Cycling Time-Trial Compared With Passive and Active Recovery in Normothermia

**DOI:** 10.3389/fspor.2021.738870

**Published:** 2021-10-25

**Authors:** Mikel Egaña, Lynn Allen, Kate Gleeson, Norita Gildea, Stuart Warmington

**Affiliations:** ^1^Department of Physiology, School of Medicine, Trinity College Dublin, The University of Dublin, Dublin, Ireland; ^2^Faculty of Science & Health, Athlone Institute of Technology, Athlone, Ireland; ^3^Institute for Physical Activity and Nutrition, School of Exercise and Nutrition, Deakin University, Geelong, VIC, Australia

**Keywords:** recovery, time trial, hydrotherapy, cycling, core temperature

## Abstract

**Background:** We investigated whether a brief cold water immersion between two cycling time trials (TT) improves the performance of the latter compared with passive and active recovery in normothermic conditions (~20°C).

**Methods:** In *Experiment 1* 10 active participants (4 women) completed two 4-km TT (Ex1 and Ex2, each preceded by a 12 min moderate-intensity warm-up) separated by a 15 min recovery period consisting of: (a) passive rest (PAS) or (b) 5 min cold water immersion at 8°C (CWI-5). In *Experiment 2*, 13 different active males completed the same Ex1 and Ex2 bouts separated by a 15 min recovery consisting of: (a) PAS, (b) 10 min cold water immersion at 8°C (CWI-10) or (c) 15 min of moderate-intensity active recovery (ACT).

**Results:** In both experiments, the time to complete the 4-km TT-s was not different (*P* > 0.05, ES = 0.1) among the trials neither in Ex1 (*Experiment 1*: PAS: 414 ± 39 s; CWI-5: 410 ± 39 s; *Experiment 2*: PAS: 402 ± 41 s; CWI-10: 404 ± 43 s; ACT: 407 ± 41 s) nor Ex2 (*Experiment 1*: PAS: 432 ± 43 s; CWI-5: 428 ± 47 s; *Experiment 2*: PAS: 418 ± 52 s; CWI-10: 416 ± 57 s; ACT: 421 ± 50 s). In addition, in all conditions, the time to complete the time trials was longer (*P* < 0.05, ES = 0.4) in Ex2 than Ex1. Core temperature was lower (*P* < 0.05) during the majority of Ex2 after CW-5 compared with passive rest (*Experiment 1*) and after CWI-10 compared with PAS and ACT (*Experiment 2*). Perceived exertion was also lower (*P* < 0.05) at mid-point of Ex2 after CWI-5 compared with PAS (*Experiment 1*) as well as overall lower during the CWI-10 compared with PAS and ACT conditions (*Experiment 2*).

**Conclusion:** A post-exercise 5–10 min cold water immersion does not influence subsequent 4-km TT performance in normothermia, despite evoking reductions in thermal strain.

## Introduction

In a variety of sporting events such as track cycling or athletic events, participants are often required to compete more than once on the same day during competitions. For instance, in track cycling, where events range from a 200 m flying sprint (lasting ~10s) to the 50 km points race (lasting ~1 h), cyclists often compete more than once per day, such as in the Omnium, a multi-race event, typically comprising of four different races held on the same day, having at times <1 h between events. These events are performed at a very high intensity and result in fatigue. Therefore, an optimal recovery strategy may play a significant role to ameliorate the decline in performance during these subsequent events. Cold water immersion (CWI) is one such strategy increasingly being employed by athletes, having been shown to be efficacious when applied between two equal bouts of high-intensity continuous or intermittent endurance exercise to elicit a superior performance in the second bout, both in normothermic (Crampton et al., 2013; Dunne et al., 2013; Mccarthy et al., 2016; Stephens et al., 2018; Egaña et al., 2019) and hyperthermic conditions (Yeargin et al., 2006; Peiffer et al., 2010). The beneficial effect of CWI appears to outperform other frequently used recovery strategies, such as contrast water therapy, active recovery or thermoneutral water immersion (Vaile et al., 2008; Crampton et al., 2013). Although the mechanisms governing the effects of CWI on subsequent performance remain to be elucidated, potential mediators include increased heat storage capacity (Kay et al., 1999; Marsh and Sleivert, 1999), increased venous return in response to the cold stimulus or hydrostatic pressure of the immersion (Wilcock et al., 2006), reactivation of cardiac parasympathetic activity (Stanley et al., 2012) and/or reduced perception of effort (Mccarthy et al., 2016).

However, the “same-day” performance effects subsequent to post-exercise CWI are still inconclusive due to methodological variations. For instance, all-out sprint cycling performance has been shown to be reduced after short-term, post-exercise CWI (Schniepp et al., 2002; Crowe et al., 2007; Crampton et al., 2014) likely owing to compromised contractile capabilities of cooled muscles (Bergh and Ekblom, 1979; Bigland-Ritchie et al., 1992; Crampton et al., 2011). By contrast, when incorporating upper-body arm-cranking exercise during lower-body CWI, all-out sprint cycling capacity in a subsequent bout is improved when compared against a lower-body CWI alone (Crampton et al., 2014). This was because core temperature (*T*_core_) was maintained during the active CWI recovery, likely enhancing the neurophysiological mechanisms that drive muscle activation compared with passive CWI. Although, therein un-immersed active recovery preserved sprint performance whereas both passive and active CWI recoveries did not (owing to CWI-induced muscle cooling and the subsequent afterdrop response). On the other hand, some studies followed CWI with extended recovery periods exceeding 1–2 h prior to the subsequent exercise bout (Versey et al., 2011; Stanley et al., 2012). Despite the applied nature of these experimental protocols (i.e., by way of simulating training and competing twice in 1 day, such as in track cycling), inter-individual influences other than water immersion (i.e., warm-down, stretching or passive rest) may affect the subsequent performance.

When the effect of post-exercise CWI has been explored on subsequent high-intensity endurance exercise performance completed immediately after the recovery protocol under normothermic conditions (~19–20°C), and thus, when recovery is not complicated by other influences, the time to failure during high-intensity constant-load exhaustive efforts is enhanced both, during cycling (Crampton et al., 2013) as well as running (Dunne et al., 2013). Furthermore, CWI evokes benefits during subsequent intermittent exhaustive high-intensity exercise (Mccarthy et al., 2016) as well as intermittent sprint protocols that mimic the typical sprint characteristics and metabolic demands of many team sport games (Egaña et al., 2019). Under hot ambient conditions (35°C), it was shown that a 5 min CWI employed after a 4-km cycling time trial (TT) significantly reduced the completion time of a subsequent 4-km TT (both TT-s preceded by a 25 min moderate-intensity exercise bout) compared with a control (passive rest) condition (Peiffer et al., 2010), while Yeargin et al. (2006) demonstrated that a post-exercise 12-min CWI improved the subsequent 2 mile running time trial.

To the best of our knowledge the effect of CWI on subsequent time trial performance has not been explored under normothermic ambient conditions when the exercise is performed immediately after the immersion. Accordingly, the aim of the present study was to compare the effects of post-exercise brief cold water immersions (5- and 10-min durations) with both passive rest and active recovery interventions on subsequent 4-km TT performance that was preceded by a 12 min moderate-intensity exercise bout. In attempting to explore the mechanistic basis of any CWI-induced effects on subsequent performance (i.e., 4-km completion time), *T*_core_, heart rate (HR) and ratings of perceived exertion were assessed. It was hypothesized that compared with passive and active recovery control conditions, CWI interventions would improve subsequent 4-km TT performance.

## Materials and Methods

### Participants

Two experiments were performed. *Experiment 1* tested the effect of post-exercise 5 min CWI on a subsequent 4-km cycling TT compared with passive recovery, whereas *Experiment 2* tested the effect of post-exercise 10 min CWI on a subsequent 4-km cycling TT compared with passive as well as active recovery.

Ten (4 women) active participants (mean ± SD; age: 21 ± 1 year; height: 178 ± 8 cm; body mass: 71 ± 11 kg, peak oxygen uptake ($\overset{∙}{\text{V}}{\text{O}}_{2\text{peak}}$): 48.1 ± 7.8 ml·kg^−1^·min^−1^, peak power (PO_peak_): 282 ± 86 W) who were accustomed to recreational cycling took part in *Experiment 1*. They visited the Human Performance Laboratory in the Department of Physiology of the Institution, on 3 days separated by at least 72 h. Thirteen different young men (mean ± SD; age: 29 ± 7 year; height: 181 ± 7 cm; body mass: 80 ± 12 kg, $\overset{∙}{\text{V}}{\text{O}}_{2\text{peak}}$: 51.2 ± 15.6 ml·kg^−1^·min^−1^, PO_peak_: 348 ± 53 W) also accustomed to recreational cycling participated in *Experiment 2*, whereby they visited the same laboratory on four separate occasions (at least 72 h apart). All participants were free from any medical conditions, (assessed by medical questionnaire and physical examination) and were non-smokers. All participants provided written informed consent prior to participation. The study was approved by the Faculty of Health Science Research Ethics Committee of the Institution and carried out in accordance with the Declaration of Helsinki.

### Experimental Protocol

#### Overview

**Figure 1:**
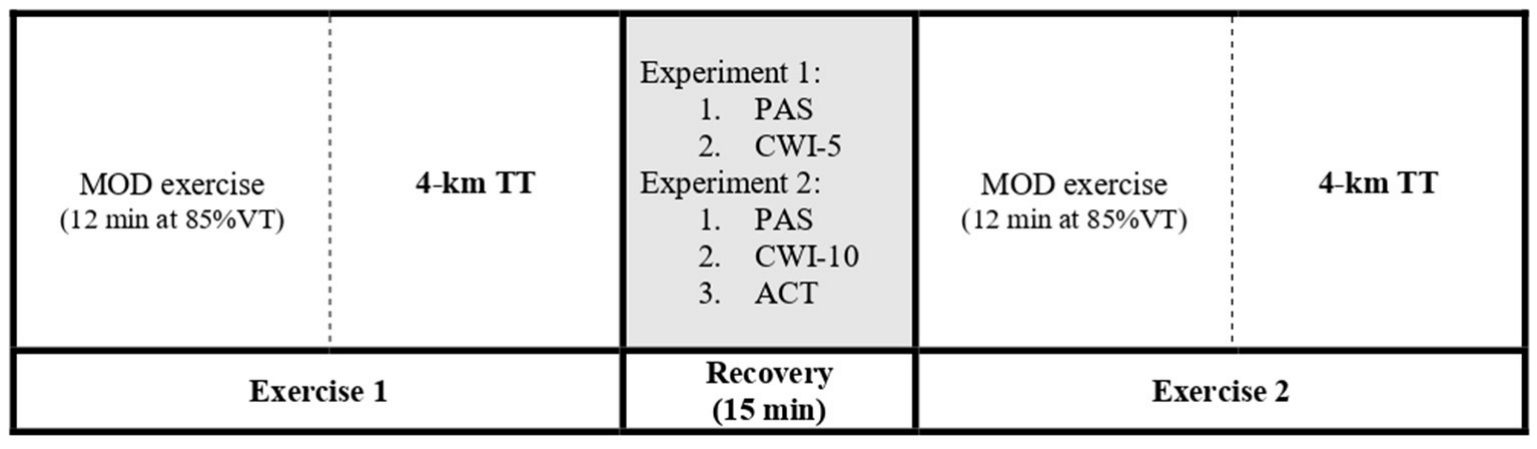
Timeline of experimental protocol. In *Experiment 1* the recovery treatments included passive recovery (PAS) and 5 min cold water immersion (CWI-5); in *Experiment 2*, passive recovery (PAS), 10 min cold water immersion (CWI-10), and active recovery (ACT). VT, first ventilatory threshold; MOD, moderate-intensity cycling exercise; TT, time trial.

An overview of the experimental protocol is shown in Figure 1. In both experimental conditions, *Experiment 1* and *2*, participants performed a preliminary incremental cycling test and familiarization to the CWI and 4-km TT test (visit 1). Thereafter, in *Experiment 1* participants were required to carry out 2 separate randomized trials (visits 2–3) separated by a minimum of 2 days, and in *Experiment 2*, three separate randomized trials separated by a minimum of 2 days (visits 2–4). Each experimental trial required the participants to complete an exercise bout (Ex1: 12 min of moderate-intensity cycling + a 4-km TT) followed by a randomized 15 min recovery period and a subsequent second identical exercise bout (Ex2).

All testing sessions within each experimental protocol were completed over a course of 7 weeks, with *Experiment 2* being carried out several weeks after *Experiment 1* was completed. Participants were asked to ensure that weekly training regimens were similar and maintained throughout this time frame. During visit 1 to the laboratory, all participants completed a 24 h food and fluid recall diary, which they were asked to replicate as closely as possible in the 24 h prior to subsequent experimental sessions. In addition, they were instructed to consume a meal consisting of approximately 200 g of carbohydrate 3 h prior to all experimental sessions. Adequate hydration status, i.e., within the accepted euhydration ranges 1,000 and 1,020, was ensured at the start of each visit by measuring urine specific gravity using an optical refractometer (Bellingham & Stanley, Hants, UK). In an effort to limit diurnal fluctuations in *T*_core_, fatigue and overall exercise capacity, all experimental sessions were held at the same time of day. Participants were asked to refrain from exercise training for at least 12 h and to avoid alcohol and caffeine consumption for 24 h prior to each visit. The experimental exercise sessions were performed in the upright position using a cycle ergometer (*Experiment 1*: Wattbike, Nottinghamshire, UK; *Experiment 2*: Excalibur Sport, Lode, Groningen, The Netherlands). In *Experiment 1* the gearing was self-selected by the participants on the Wattbike during the practice trial and then replicated during each TT. In *Experiment 2* the Lode ergometer was set to linear mode so that with increasing pedaling rate the work rate increased. During all experimental sessions the ambient temperature of the laboratory wherein both the exercise and recovery protocols were carried out was held constant (20 ± 1°C). In addition, during the exercise protocols, participants were cooled with a 300-mm diameter fan (Micromark, UK) placed 1 m in front of them that produced an air flow equivalent to 3 km.h^−1^.

#### Graded Incremental Test and Familiarization

All participants performed a graded incremental test to failure to determine $\overset{∙}{\text{V}}{\text{O}}_{2\text{peak}}$ and the first ventilatory threshold (VT) on an upright cycling ergometer (for both experiments: Excalibur Sport, Lode, Groningen, The Netherlands). After a 3 min period of rest in a seated position, the test commenced with participants cycling at 30W for 1 min, with incremental increases of 20W (women) or 30W (men) every min until task failure. The VT was determined using the V-slope method (Beaver et al., 1986). After the graded incremental test, participants were familiarized with the constant-load bout and 4-km TT cycling protocol and cold water immersions.

#### Experimental Trials

##### Exercise Bouts (Ex1 and Ex2)

Each of the experimental sessions was comprised of two identical exercise protocols (Ex1 and Ex2) separated by a 15 min recovery interval. The exercise protocol consisted of a 3 min “baseline” cycling period at 10W followed by a 12 min constant load cycle at 85% of each participants VT, a 2 min seated rest period, and finally a 4-km cycling TT. A 4-km TT test was chosen because it has been shown to be a reproducible test (Stone et al., 2011). Participants were instructed to complete the 4-km cycling TT in as fast a time as possible whilst receiving visual feedback for distance completed, but all were blinded to their exercise times. The 12 min moderate-intensity constant-load bouts, which were initiated from a 3 min baseline at 10W, were carried out to warm up the active musculature.

##### Recovery Interventions

All recovery interventions were performed in a balanced randomized order throughout the study. On each testing day of *Experiment 1*, one of the following recovery interventions were performed: (a) passive un-immersed rest in a seated position (PAS) and (b) 5 min CWI at 8°C (CWI-5). On testing days of *Experiment 2* the following recovery interventions were performed: (a) PAS, (b) 10 min CWI at 8°C (CWI-10) and (c) active recovery (ACT) comprising cycling at 40% VO_2peak_.

Between min 5 to 10 of the CWI-5 trial, participants were immersed in a custom-built bath (Sturdy Products, Co. Wicklow, Ireland) positioned next to the cycling ergometer. Five min periods of transition from the ergometer to the bath, as well as from the bath to the ergometer were allowed. These transition times however were shortened in CWI-10 testing trials to 2.5 min to allow for the longer immersion time where participants were immersed between 2.5 and 12.5 min. During the transitioning period to the bath, participants removed their cycling shoes, training top, shorts and socks and changed into swimwear. During the second transition, participants were provided with towels to dry themselves prior to redressing for Ex2. Any surplus time during the transition periods was spent passively resting in a seated position on a stool. During the recovery treatments, participants remained upright in a seated position, with their backs placed firmly against the posterior wall of the bath and feet against the anterior wall with their knees flexed (~90°) to ensure full leg immersion. During the passive condition, participants sat in this same position, albeit in an empty bath. During the periods of immersion, the water remained relatively stagnant, and at approximately sternal level for each participant to evoke a significant muscle (and core) cooling effect, without drastically influencing core temperature, as can occur with deeper (i.e., neck level) immersion (unpublished observations). The 8°C water temperature utilized herein was selected given its frequent application in water immersion protocols for recovery post-exercise (Wilcock et al., 2006). Furthermore in similar normothermic conditions, in comparison to passive rest, cold water immersion at 8°C has been shown to better enhance subsequent sustained running performance than with cold water immersion at 15°C (Dunne et al., 2013). The 5 min CWI was chosen because it has previously been shown to evoke significant benefits in hyperthermic conditions during subsequent 4 km TT, as well as in normothermic conditions during brief (6–8 min) intermittent exhaustive high-intensity exercise protocols (Mccarthy et al., 2016) and prolonged (40 min) intermittent sprint protocols designed to mimic activity patterns of team-sports (Egaña et al., 2019). The 10 min duration was chosen to explore whether it would induce a larger reduction in thermal and cardiovascular strain (compared with 5 min CWI) and subsequent superior 4 km TT performance. The water temperature was monitored with a 6000 series bench thermometer (TM Electronics Ltd., West Sussex, UK) with a type T thermocouple, and ice was added to decrease the temperature when needed. In order to limit potential bias in treatment response, participants were neither informed of expected outcomes of each recovery intervention, nor had information in relation to the belief effect of each intervention collected before the study.

### Measurements

#### Core Temperature

During each testing session *T*_core_ (gastrointestinal temperature) was recorded continuously using ingestible body temperature sensors swallowed with tepid water approximately 6 h prior to testing, and a hand held data receiver (CorTemp, HQ, Florida, USA). This method provides a valid index of core temperature in comparison with esophageal temperature, the best available index for core temperature in exercise studies (Byrne and Lim, 2007).

#### Pulmonary Gas Exchange, Heart Rate, and Ratings of Perceived Exertion

During the incremental cycling tests, participants wore a facemask to continuously collect expired air using an online metabolic system (Cosmed Quark CPET, Rome, Italy), and HR was recorded on a second-by-second basis (S610i, Polar Electro Oy, Finland). Prior to, and at the end of each 12 min constant-load bout and 4-km TT exercise protocol, ratings of perceived exertion were documented in relation the Borg scale (6–20) (Borg, 1990).

### Statistical Analysis

Data are presented as mean ± SD. Two-way repeated measures ANOVA (trial by time) was used to analyse the 4-km TT times to failure, HR, *T*_core_ and ratings of perceived exertion responses within each experiment. Differences were detected using Holm-Sidak *post*-*hoc* tests. Sphericity was assessed and adjusted where the assumption of sphericity could not be assumed (ε > 0.75 = Huynh–Feldt; ε < 0.75 = Greenhouse–Geisser). Significance was set at *P* ≤ 0.05. Effect sizes (ES) were also calculated using Cohen's *d* to compare the magnitude of the difference in time to completion between the trials (Cohen, 1988). Thresholds for effect sizes were set as the following: <0.19, trivial; 0.20–0.49, small; 0.5–0.79, moderate; >0.8, large; with an effect size of 0.2 being considered as the smallest worthwhile positive effect. Effect size was computed as d = [(mean Ex1 – mean Ex2) / pooled standard deviation]. Within-participant consistency of time trial performance during Ex1 was established using the coefficient of variation (CV) derived from the log-transformed data using the Excel spreadsheet of Hopkins (Hopkins, 1997). A power analysis indicated that nine participants per group were required to detect a ~ 4% improvement in 4 km TT with a power of 0.80 and alpha of 0.05 for an ANOVA calculation design based on 3 trials. This was estimated using means and standard deviations from previously published similar studies (Peiffer et al., 2010; Tomazini et al., 2020).

## Results

### 4-km Cycling Time Trial Performance

**Figure 2:**
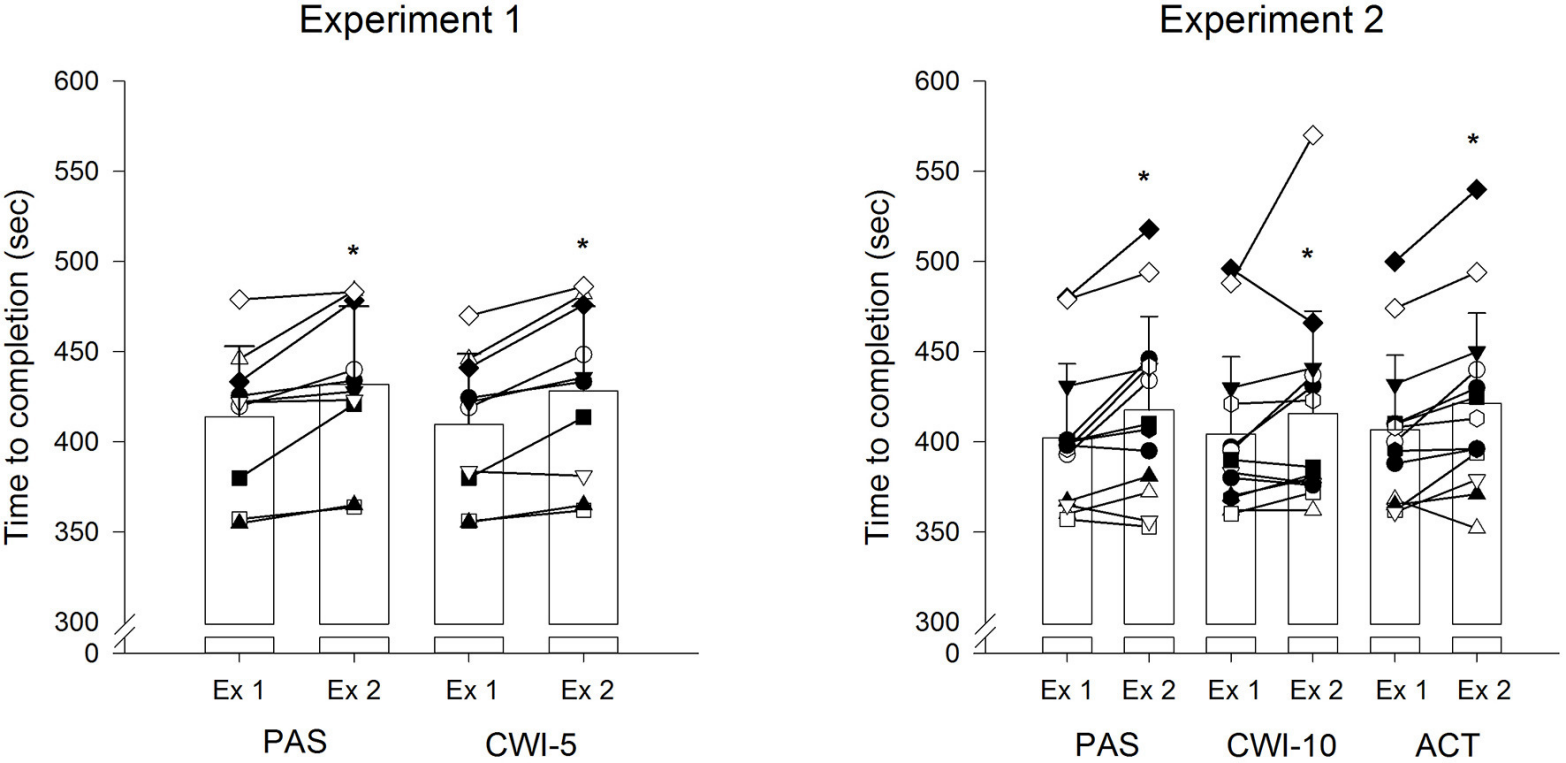
Individual and mean (±SD) cycling completion times for the 4-km time trials (TT) during Exercise 1 (Ex 1, pre-recovery) and Exercise 2 (Ex 2, post recovery) for the experimental conditions. PAS, passive recovery; CWI-5, 5 min cold water immersion; CWI-10, 10 min cold water immersion; ACT, active recovery. *Significantly different from Ex1 (*P* < 0.05).

*In Experiment 1*, the time to complete the 4-km TT in both trials was not different (*P* > 0.05, trivial ES) in both, Ex1 (PAS: 414 ± 39 s; CWI-5: 410 ± 39 s) and Ex2 (PAS: 432 ± 43 s; CWI-5: 428 ± 47 s). In both conditions, the time to complete the time trial was longer (*P* < 0.05, small ES) in Ex2 than Ex1 (Figure 2).

Similarly, in *Experiment 2*, the time to complete the 4-km TT in the 3 trials was not different (*P* > 0.05, trivial ES) in both, Ex1 (PAS: 402 ± 41 s; CWI-10: 404 ± 43 s; ACT: 407 ± 41 s) and Ex2 (PAS: 418 ± 52 s; CWI-10: 416 ± 57 s; ACT: 421 ± 50 s). In all conditions, the time to complete the time trials was longer (*P* < 0.05, small ES) in Ex2 than Ex1 (Figure 2).

### Core Temperature

**Figure 3:**
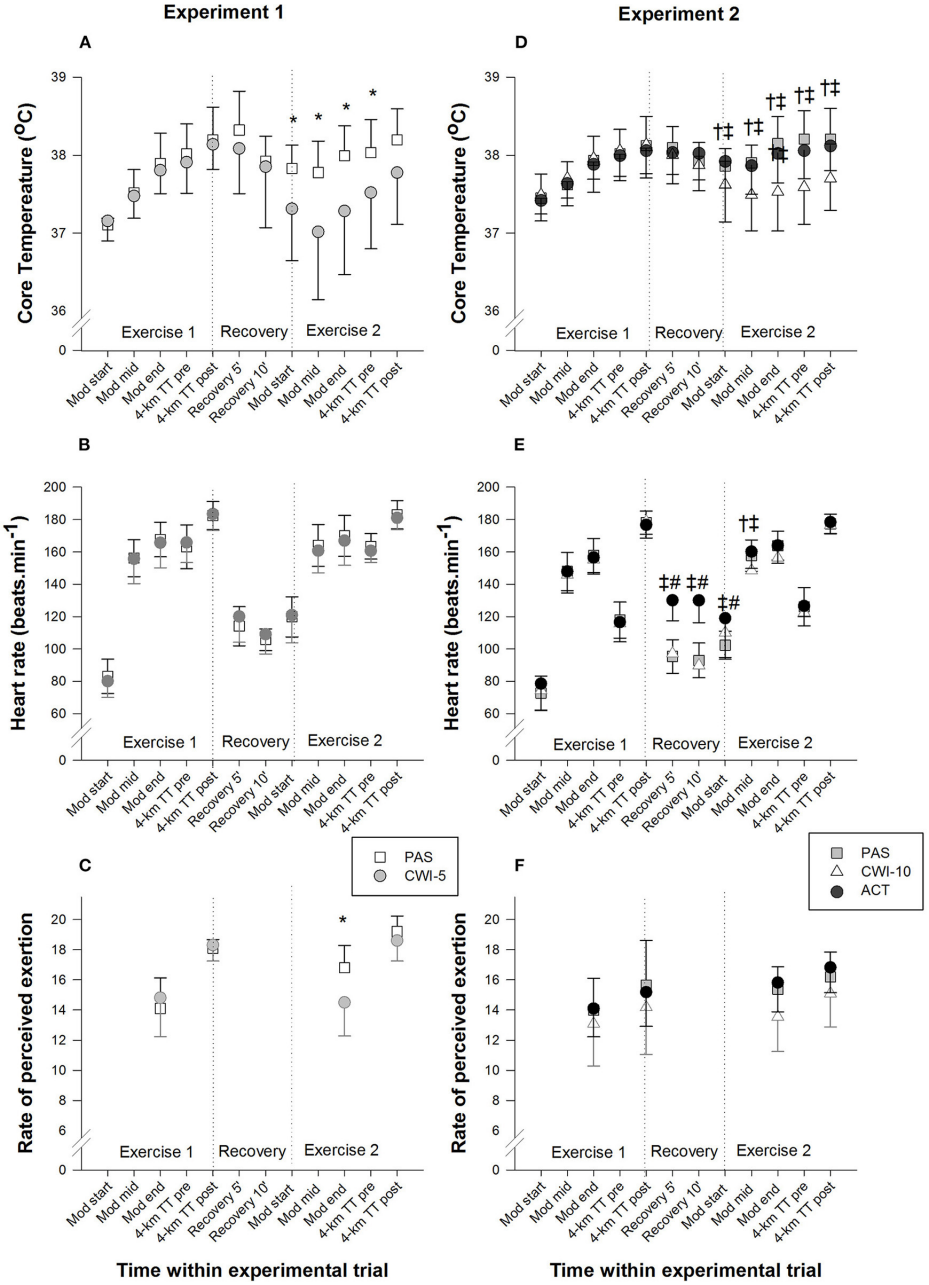
Mean (±SD) core temperature (**A**, *Experiment 1*; **D**, *Experiment 2*), heart rate (**B**, *Experiment 1*; **E**, *Experiment 2*) and ratings of perceived exertion (**C**, *Experiment 1*, **F**, *Experiment 2*) responses at different time points during the experimental conditions. PAS, passive recovery; CWI-5, 5 min cold water immersion; CWI-10, 10 min cold water immersion; ACT, active recovery; Mod, 12-min moderate-intensity constant-load exercise; mid, mid time point; TT, time trial. *CWI-5 significantly different from PAS (*P* < 0.05). ^†^CWI-10 significantly different from PAS (P < 0.05). ^‡^CWI-10 significantly different from ACT (*P* < 0.05). ^#^Active recovery significantly different from PAS (*P* < 0.05).

*T*_core_ responses across all conditions over time are presented in Figure 3A (*Experiment 1*) and Figure 3D (*Experiment 2*). During all time points of Ex,1 *T*_core_ values were similar between conditions in both experiments. *T*_core_ progressively increased until the end of Ex1 by a magnitude that was not different among the conditions (*Experiment 1*: PAS: 38.2 ± 0.4°C; CWI-5: 38.1 ± 0.3°C; *Experiment 2*: PAS: 38.1 ± 0.4°C; CWI-10: 38.1 ± 0.4°C; ACT: 38.1 ± 0.4°C). *T*_core_ then decreased during the 15 min recovery phase with no significant differences among conditions. At the onset of the constant-load bout immediately after the recovery period and until the beginning of the 4-km time trial, CWI-5 evoked significantly lower (*P* = 0.02) *T*_core_ responses compared with the passive rest trial (trial × time interaction, *P* = 0.03) in *Experiment 1* (Figure 3A). In *Experiment 2*, during the entire period of Ex2, CWI-10 evoked significantly lower (*P* < 0.001) *T*_core_ responses compared with the passive and active recovery trials (trial × time interaction, *P* < 0.01) (Figure 3D).

### Heart Rate

There was no significant difference in HR between the experimental conditions within both experiments during Ex1 (*Experiment 1*: Figure 3B; *Experiment 2*: Figure 3E). In *Experiment 1*, there was a tendency for HR to be lower during CWI-5 than PAS during Ex2 (trial × time interaction, *P* = 0.09), however, it did not reach statistical significance (Figure 3B). In *Experiment 2*, there was a trial × time interaction (*P* < 0.01) where ACT recovery evoked a larger (*P* < 0.01) HR compared with PAS and CWI-10 conditions during the recovery period and onset of the subsequent constant-load bout; and at mid-point of the constant-load bout HR was also larger (*P* = 0.01) during ACT compared with CWI-10 as well as PAS compared with CWI-10 conditions. HR responses were similar among conditions during the 4-km time trials during Ex2 (Figure 3E).

### Rating of Perceived Exertion

In *Experiment 1*, ratings of perceived exertion were significantly lower (*P* < 0.001) at the end of the moderate-intensity cycling bout of Ex2 during CWI-5 compared with PAS rest (trial × time interaction, *P* = 0.001) (Figure 3C). In *Experiment 2*, CWI-10 evoked lower ratings of perceived exertion than PAS and ACT conditions (main effect, trial; *P* = 0.001), but there was no group × time interaction (*P* = 0.3) (Figure 3F).

## Discussion

The main finding of the present study, in contrast with our principal hypothesis, was that the cold water immersion interventions did not improve the completion time during the subsequent 4-km cycling TT compared with PAS or ACT recovery interventions. Both CWI treatments elicited reductions in *T*_core_ (and most likely muscle temperature) responses during the second exercise bout, however, herein, these adaptations did not evoke performance benefits.

Our findings are in contrast to similar previous studies that reported benefits in times to exhaustion during both, constant-load (Crampton et al., 2013; Dunne et al., 2013), and intermittent high-intensity exercise in normothermia (Mccarthy et al., 2016), as well as in time trial performance in hyperthermic conditions (Yeargin et al., 2006; Peiffer et al., 2010) when exercise was performed immediately following a short CWI when compared with passive and/or active recovery. In agreement with the present findings, Stephens et al. (2018) showed no difference in post-CWI 4-min time trial performance compared with a passive control condition, however, the time trial was carried out more than 40 min after the recovery interventions.

Herein, a significant afterdrop (hypothermic undershoot) effect was elicited (~0.3°C), immediately after each water immersion intervention, likely attributed to a rapid dissipation of blood from the cooled peripheral tissues to the core (Arborelius et al., 1972; Mittleman and Mekjavic, 1988; Bristow et al., 1994). These afterdrop effects are consistent with previous studies that explored the effects of CWI employed between two equal high-intensity exercise protocols carried out immediately before and after the CWI recovery period under normothermia where the *T*_core_ achieved at the end of the pre-recovery exercise bout (Ex1) was similar (~38–38.5°C) (Crampton et al., 2013; Dunne et al., 2013; Mccarthy et al., 2016; Egaña et al., 2019). Interestingly, during the 15 min recovery period, CWI-5 induced a larger absolute drop in *T*_core_ (~0.8°C) compared with CWI-10 (~0.5°C) which is consistent with findings reported by Mccarthy et al., 2016 under similar conditions. This is likely because some participants showed an exaggerated drop in *T*_core_ during the CWI-5 conditions (and hence, the variation for this variable for this condition was larger than for the other conditions, see Figure 3A), whereas some participants during the CWI-10 trial reported shivering which could have induced increases in metabolic heat production and *T*_core_. Consequently, CWI-10 (as was the case for CWI-5) did not induce any ergogenic effects compared with PAS and ACT interventions (trivial effect sizes in all conditions), suggesting that the duration of the CWI, within a 15-min recovery phase, does not affect the completion time of a subsequent 4 km time trial in normothermia.

That the observed drop in *T*_core_ herein was accompanied by reductions, albeit non-significant, in HR during the 12-min constant-load bouts, suggests CWI reduced thermal and cardiovascular strain (Parkin et al., 1999; Marino, 2002). Nevertheless, it is noteworthy that this prolonged reduction in *T*_core_, negatively impacts the contractile apparatus of cooled muscles (Bergh and Ekblom, 1979; Bigland-Ritchie et al., 1992) particularly during maximal efforts. Specifically, individuals performing all-out cycling bouts (i.e., 30 s “Wingate” sprints) immediately after CWI, demonstrate significant reductions in performance when compared with passive or active recovery conditions (Schniepp et al., 2002; Crampton et al., 2014). Indeed, when an intermittent sprint protocol that replicates the typical sprint characteristics of many team sport games (20–30 brief, 6–8 s, all-out sprints separated by 14–120 s active recovery periods within a 40 min “half-time”) was carried out immediately after CWI, the total work done and power output during the early sprints was reduced, while later sprints showed higher work and power output, leading to an overall significant increase in the second-half sprint performance (Egaña et al., 2019). As such, it is likely that once muscles are adequately warmed up, the increased heat storage capacity facilitated by the lower *T*_core_ response contributed to the improvements observed in the subsequent sprint performance (Lee and Haymes, 1995; Booth et al., 1997; Kay et al., 1999). In line with this notion, participants performing 3 × 4 min high-intensity efforts after CWI showed a likely higher anaerobic contribution (at least partly due to a reduced perfusion in cooled muscles) during the first bout (compared with passive rest), but the anaerobic contribution was less likely to be different between CWI and PAS conditions in the three remaining bouts (Stanley et al., 2014).

For that reason, in the present study participants performed a 12-min moderate-intensity warm-up prior to carrying out the 4-km TT bouts. Importantly, Mccarthy et al. (2016) used the same CWI recovery as well as post-recovery warm-up protocol and observed that the time to failure during an intermittent high-intensity cycling protocol lasting ~6–8 min (i.e., similar duration as the times to complete the 4-km TT in the current study) was significantly improved compared with passive rest. In addition, using longer (i.e., 15–30 min) CWI periods and a similar post-recovery warm-up, the time to failure during a subsequent constant-load exercise was also significantly prolonged during both, running (exercise bouts lasting ~20–27 min) (Dunne et al., 2013) and cycling (exercise bouts lasting ~10–20 min) (Crampton et al., 2013) compared with passive rest. This would suggest that CWI is more efficient at enhancing exhaustive intermittent and/or constant-load efforts compared with time trial efforts, at least in normothermic conditions. It is worth noting, however, that Peiffer et al. (2010) reported CWI-induced benefits in subsequent 4-km TT-s in hyperthermia, where participants completed a longer (25 min) moderate-intensity exercise bout prior to the 4-km TT-s. Thus, we cannot exclude the possibility that a longer warm-up period is needed to adequately warm up the active muscles and maximize subsequent 4 km TT performance.

In the present study active recovery did not induce any improvements in cycling TT performance compared with PAS and CWI recoveries. While active recovery is one of the most frequently employed recovery interventions in sports, given, among other factors, its ability to accelerate lactate clearance (Bangsbo et al., 1994); its effectiveness regarding subsequent exercise performance is inconclusive. For instance, the effectiveness of active recovery and CW1 appear to be on a par in maintaining the endurance performance of eccentric exercises, well-known to induce a substantial degree of muscle damage and high lactate levels, such as “lead” style climbing (Heyman et al., 2009). However, active recovery appears less effective than CWI in maintaining time to exhaustion during concentric non-damaging submaximal exercise akin to that employed herein (Crampton et al., 2013).

## Limitations

A number of limitations of the present study must be acknowledged. First, our study participants cycled recreationally, and thus, day to day variation may be greater than in trained cyclists. However, all participants performed at least one familiarization trial which helped reduce any potential learning effect. Indeed, the overall coefficient of variation across the Ex1 time trials herein was low (~3.2%) yet still higher than that reported for trained cyclists (~1.9%) (Stone et al., 2011). Nevertheless, the involvement of recreational active participants in the present study, limits the applicability of the results to athletic settings. Second, we acknowledge that it would have been preferable if the same participants completed all recovery interventions herein. For practical reasons this was not feasible. *Experiment 1* was designed to mimic the study protocol (i.e., comparing CWI-5 vs. PAS conditions) used by Peiffer et al. (2010) under hot ambient conditions (35°C), albeit in normothermia. However, upon observation that a 5 min CWI stimulus was not sufficient to induce benefits in subsequent TT performance, several weeks later in *Experiment 2* we explored whether a longer 10 min CWI would be more effective (than CWI-5) on subsequent TT in different participants, while also including a commonly used active recovery intervention. Third, the two experiments herein used different cycling ergometers, while it would have been preferable if they used the same one. This was not feasible also for practical reasons (i.e., the Wattbike was not available to use in *Experiment 2*). Despite this, both the Lode (Driller, 2012) and Wattbike (Bellinger and Minahan, 2014) cycling ergometers have been shown to be reliable for TT performance, so, it is unlikely that this would have affected the present study outcomes. Finally, we acknowledge that additional physiological parameters such as muscle temperature, muscle perfusion, blood lactate or $\overset{∙}{\text{V}}{\text{O}}_{2}$ kinetics measures would have been helpful to further explain the present results and demonstrate whether the warm-up period prior to the second TT was of sufficient duration to warm up the active muscles.

## Conclusion

In conclusion, the present study showed that in comparison to passive and active recovery strategies, a brief (5–10 min) post-exercise cold water immersion at 8°C does not improve the performance during a subsequent 4-km cycling time trial. Our findings do not support the use of cold water immersion in between two time trial efforts lasting approximately 7 min when preceded by a 12 min moderate-intensity warm-up in normothermic ambient conditions. Further studies should assess whether CWI affects subsequent sustained high-intensity efforts when these efforts are preceded by longer warm-up periods to ensure active muscles are suitably warmed up.

## Data Availability Statement

The raw data supporting the conclusions of this article will be made available by the authors, without undue reservation.

## Ethics Statement

The studies involving human participants were reviewed and approved by Faculty of Health Science Research Ethics Committee, Trinity College Dublin. The patients/participants provided their written informed consent to participate in this study.

## Author Contributions

ME, SW, LA, and KG conceived and designed the research and analyzed data. LA and KG conducted experiments and collected all participant data. ME drafted the paper. ME, SW, and NG interpreted data and contributed to the writing of the final paper. All authors contributed to the article and approved the submitted version.

## Conflict of Interest

The authors declare that the research was conducted in the absence of any commercial or financial relationships that could be construed as a potential conflict of interest.

## Publisher's Note

All claims expressed in this article are solely those of the authors and do not necessarily represent those of their affiliated organizations, or those of the publisher, the editors and the reviewers. Any product that may be evaluated in this article, or claim that may be made by its manufacturer, is not guaranteed or endorsed by the publisher.
